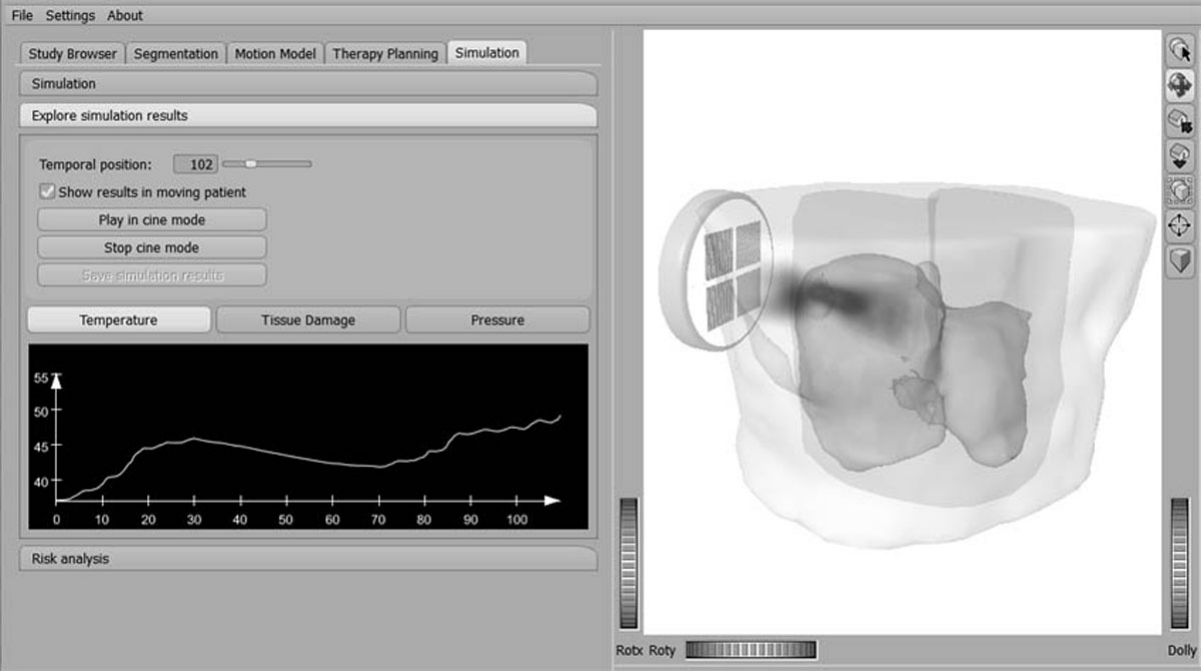# Accounting for sliding motion in fast numerical simulations of abdominal HIFU applications for targets under respiratory motion

**DOI:** 10.1186/2050-5736-3-S1-P51

**Published:** 2015-06-30

**Authors:** Michael Schwenke, Jan Strehlow, Sabrina Haase, Christine Tanner, Yoav Levy, Tobias Preusser

**Affiliations:** 1 Fraunhofer MEVIS, Bremen, Germany; 2 ETH Zurich, Zurich, Switzerland; 3 InSightec Ltd, Tirat Carmel, Israel; 4 Fraunhofer MEVIS/Jacobs University Bremen, Bremen, Germany

## Background/introduction

Non-invasive ablation and hyperthermia treatments of abdominal organs using High Intensity Focused Ultrasound (HIFU) still impose severe challenges. Especially for liver and kidney treatments, the target motion and accessibility over the entire respiratory cycle make the application of HIFU difficult. To allow for a safe, effective, and efficient treatment, detailed planning and monitoring of the intervention is needed. Current general-purpose physics simulation software cannot fulfill the real-time requirements of the therapy-monitoring application. Our goal is to improve this by numerical simulation to allow for fast and accurate treatment planning and real-time detection of motion-induced risks during HIFU-therapy.

## Methods

We developed a method to numerically simulate HIFU using patient-specific motion information in real time. The bio-heat equation describing the temperature distribution in the patient is mathematically transformed to a static reference anatomy. The numerical solution can then be performed in the reference anatomy on a static computational domain. Patient-specific respiratory motion information is provided by an abdominal motion model that can be fed with sparse tracking data in the clinical setting. The respiratory motion is modelled allowing sliding motion of the inner organs along the abdominal wall.

Motion information is fed into the simulation by an unstructured moving point cloud. On top of this point cloud a tetrahedral mesh is build to interpolate the motion information. The mesh is split at the abdominal wall into individually moving parts to allow the sliding motion of the liver along the abdominal wall. The numerical simulation thereby resolves the sliding boundary and allows the prediction of the temperature rise also in the beam path between transducer and target. Fast US beam steering is simulated using ultrasound pressure field pre-computations for key states over the respiratory cycle. Any remaining location mismatch between closest pre-computation and actual target location is compensated using an additional transformation. The numerical simulation method is integrated into a HIFU-therapy planning tool. Based on image data the target volume, anatomical and risk structures are defined. Treatment planning is then performed guided by direct feedback from the numerical simulation including respiratory motion of the domain. Using the feedback from the simulation, the user can manually optimize transducer and target location and HIFU parameters.

## Results and conclusions

The method accounts for patient-specific anatomy and motion information in real time. Sliding motion of the inner organs along the abdominal wall is accounted for by the simulation. The transformation to the static reference frame results in an accurate scheme for long simulation durations. The developed method can be a possible important building block for HIFU-therapy planning and conduction in moving organs.

**Figure 1:**